# Bioimage informatics: Investing in software usability is essential

**DOI:** 10.1371/journal.pbio.3002213

**Published:** 2023-07-31

**Authors:** Perrine Paul-Gilloteaux

**Affiliations:** 1 Nantes Université, CHU Nantes, CNRS, Inserm, BioCore, US16, SFR Bonamy, Nantes, France; 2 Nantes Université, CNRS, Inserm, l’Institut du Thorax, Nantes, France

## Abstract

In 2018, *PLOS Biology* announced CellProfiler 3.0, which has become one of the most used pieces of image analysis software in biology. The rapid adoption of this software speaks to the importance of user experience to disseminate new methods of bioimage informatics.

## Abstract 2

In 2018, PLOS Biology announced CellProfiler 3.0, which has since become one of the most used pieces of image analysis software in biology. This Perspective looks at the past 20 years of bioimage informatics and highlights the importance of software usability for diffusing new methods within the field.

This article is part of the *PLOS Biology* 20th Anniversary Collection.

Microscopy has drastically evolved over the past few decades, pushing back the frontiers in all dimensions and bringing new insights about the organization of biological processes in space and time. Microscopy is no longer an observational tool, but a quantitative tool for biology. This progress has been made as a result of collaborative efforts between the fields of biology, physics, and chemistry, but also computer sciences. Nowadays computational methods are one of the components of most microscopic acquisitions (e.g., super-resolution microscopy) and are required to extract quantitative content from these images. Bioimage informatics involves the development of these methods and software for processing, analyzing, managing, and visualizing cellular and molecular imaging data [1].

The premises of bioimage informatics were set as early as the 1960s, but even in the 1990s, automation of biological object recognition was met with great skepticism [2]. Bioimage informatics is now recognized as an essential tool for biological research that relies on light or electron microscopy. As shown in Fig 1, it was only in the 2000s that researchers working on developing computer vision methods for microscopy systems in biology started to openly self-affirm as a separate group [1,3], having originally been associated with the medical image processing community. Bioimage informatics was still considered a nascent field in 2012 [4], driven by progress in genomics that called for scalable phenotyping of cells and by supporting analysis of new microscopy instrumentation and methods. It has also pushed the imaging process towards higher resolution (e.g., deconvolution or single-particle localization techniques), higher speed, and longer acquisitions (e.g., denoising), among other advances.

**Fig 1:**
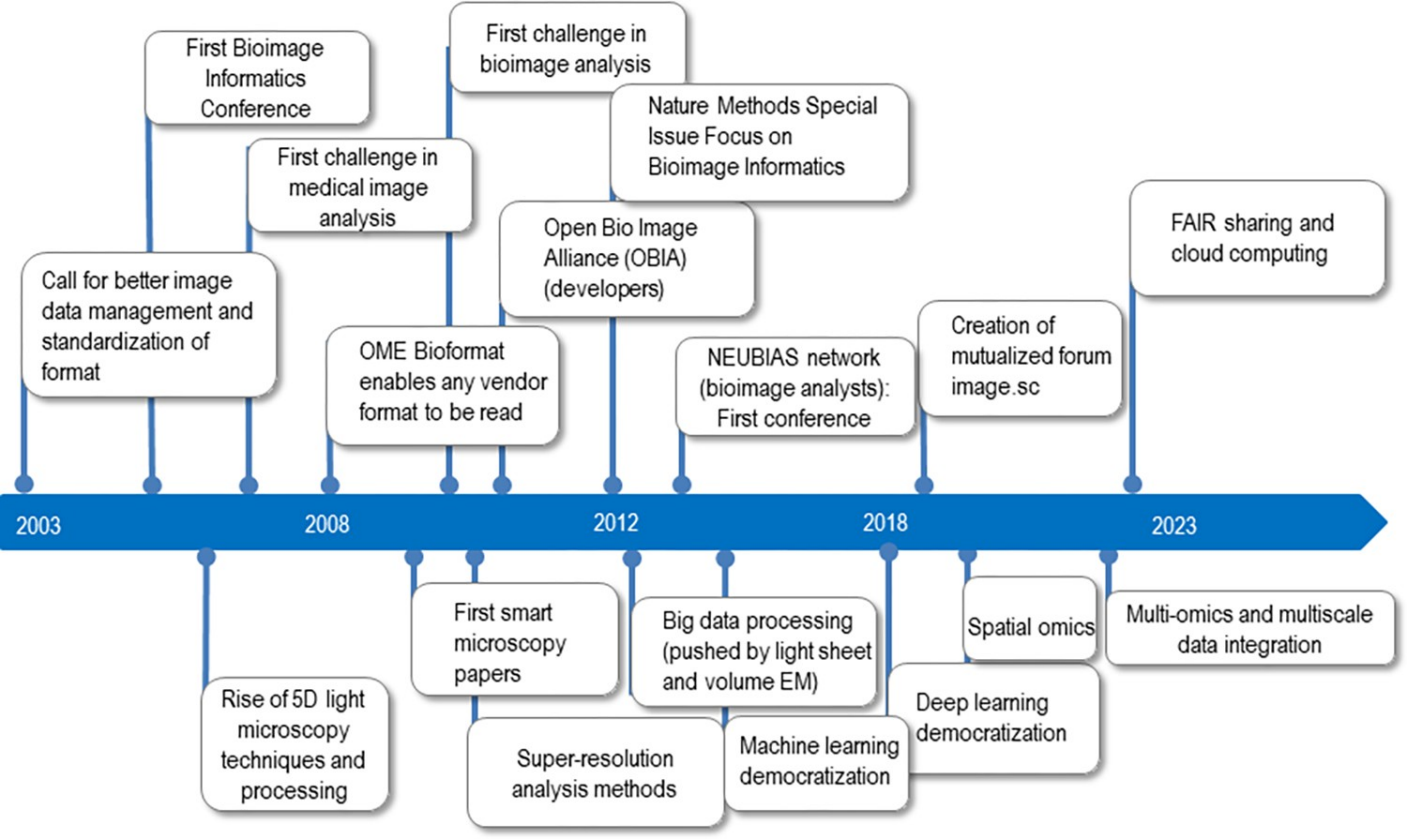
Timeline of key events in the past 2 decades of bioimage informatics. This timeline highlights key events that have contributed to the current status of bioimage informatics as an indispensable tool in biological research involving bioimaging. The events presented are based on the author’s perspective and bias. While the history of bioimage informatics started earlier, notably with ImageJ in 1987 for NIH ImageJ and IMOD in 1996, the timeline covers the years from 2003 to the present day. It includes milestones such as the development of open-source software for image analysis, the establishment of standardized file formats, and the emergence of deep learning–based approaches, as well as the construction of the community. This timeline is not meant to be comprehensive but rather to provide a simplified overview of the major events in bioimage informatics during this period. Note that due to reference limitations, no citations are provided. EM, electron microscopy; FAIR, findable, accessible, interoperable, and reusable; NEUBIAS, Network of EUropean BioImage AnalystS; OME, Open Microscopy Environment.

Over the past decade, the landscape of bioimage informatics has evolved and some key free and open-source platforms have proved their usefulness to the biosciences community [5]. The success of these platforms has relied not only on the quality of the methods implemented but also on the user interface and user experience, ease of installation, shallowness of the learning curve, presence of guidance and examples, availability of training, and a reactive community.

One such platform is CellProfiler, which was initially published in 2006 for high-throughput screening of images and to detect cell phenotypic consequences of different types of perturbations [6]. The interface allows users to construct their image analysis pipeline easily without requiring coding skills, test each step of the pipeline with visual feedback, and move to batch processing effortlessly. The first version was limited to 2D images. In 2018, the release of version 3.0 in *PLOS Biology* brought several key improvements [7]. It added the ability to process 3D data sets, switched to using the coding language Python, thereby giving users access to more open-source libraries to extend its functionalities, and provided architecture adapted to cloud computing. Furthermore, it was one of the first software platforms to pave the way for deep learning integration, allowing users to apply existing models or train new ones.

Software is an essential component of bioimage informatics, enabling researchers to access and utilize newly developed methods and algorithms. However, software is more than just a gateway to accessing these methods; it is also crucial for disseminating these methods to a broader audience. From a user perspective, a new investigation comes with the need to fine-tune parameters of workflows, and the power and impact of CellProfiler comes from its usability for this purpose. The minimal criteria for usability that software should meet to ensure it has a broad impact have already been discussed [8], criteria that are easily met by CellProfiler [6,7]. It has to be underlined that even the best methods will have limited or no impact in the life science community if they are not integrated into an easy-to-use, well-documented, and exemplified software platform. An illustrative example of this is the outcomes of the challenges organized by the bioimage informatics community. In these challenges, organizers define a specific task such as cell tracking, deconvolution, or nuclei segmentation and provide data sets and metrics to benchmark candidate methods [9]. When looking at the leader boards, one can see that the methods that perform the best are rarely used further in biological applications. The main reason is that they are usually not easily accessible to the end user. They often have software for implementation, but it is not usable because of a lack of interface and guidance or because of the complexity of the parameters used.

The effort required to develop software usability takes time and resources but is not recognized by the academic world, including funders, as a scientific output. These efforts have to be shared between bioimage informaticians and biologists or bioimage analysts who are the software users. Software users can help by acknowledging the use of implemented methods instead of only citing the main software platform’s reference paper. They also need to understand the main principles of the methods implemented in the software in order to apply them correctly. For this purpose, good teaching materials should explain not only how to use the software but also how the methods work and under which assumptions. Advertising and getting feedback on how to use methods is also important; the Image.sc forum is a great venue for that [10], as well as repositories of bioimaging software such as BioImage Informatics Index. Bioimage informaticians should also better recognize the providers of biological data and problems in the publications in their fields, and the efforts made to test or produce tutorials on software usage.

New challenges for bioimage informatics are arising from the usability side. The transition to deep learning and the use of particular hardware and deep learning libraries has caused software installation to become more difficult. Efforts are underway to improve access to deep learning, both locally from the biologist’s computer [11] but also via virtualization and cloud computing. In a similar vein, the open data revolution [12] should be applied to processing: Bioimage processing needs to be FAIR (findable, accessible, interoperable, and reusable), whether you are sharing pretrained deep learning models or developing workflows, for which sharing code is only a first step [13].

Beyond the usability of software, bioimage informatics is facing new methodological challenges, in particular those related to the integration of data extracted from images with other “omics” data, and the rise of deep learning algorithms. New statistical methods for analysis and visualization are needed to deal with the large quantities of data involved. One of the challenges from this side is the ability to provide the uncertainty attached to data extracted from images, in the sense of the level of confidence of quantitative measurements. Another challenge for the field that affects both methods and software development is how to facilitate the democratization of smart microscopy technology, in which the immediate processing of acquired images is used to adapt the acquisition accordingly. This will require bioimage informaticians to work even more closely with hardware, not just software.

Bioimage informatics is now an established field that relies on developments in image analysis methods but also those in software and computer science. The success of CellProfiler [6,7] shows how investment in software usability can lead to a high impact in the life sciences and the importance of supporting it.
